# Effect of Food Mass in the Gut on the Metabolic Rate of *Carcinus maenas* in the Field

**DOI:** 10.1002/ece3.71614

**Published:** 2025-06-24

**Authors:** David L. Neu, Laura S. Fletcher, Mikayla Bolander, Vibalia Raj, Bailey N. Marlow, Blaine D. Griffen

**Affiliations:** ^1^ Biology Department Brigham Young University Provo Utah USA

**Keywords:** body mass, crustacean, energetics, European green crab, specific dynamic action

## Abstract

Numerous laboratory studies have shown that meal size can influence the metabolism of individual organisms. However, in nature, meal size can vary simultaneously with a host of other factors that are often controlled under experimental conditions (e.g., reproductive state, health or physiological condition, temperature, meal composition, etc.). This study examines the influence of the mass of food in the gut (i.e., a proxy for meal size) on the metabolic rate of the European green crab ( *Carcinus maenas* ) in the field when other factors are not controlled in order to examine the relative influence of meal size on postprandial metabolism (i.e., specific dynamic action or SDA) compared to other factors known to influence metabolism. We collected 383 crabs intertidally during low‐tide periods and measured their metabolic rates in situ, followed by dissection to assess the mass of food in the gut, as well as reproductive and body condition metrics. We found that metabolic rate increased with body mass and with the mass of food in the gut, and decreased with gravid individuals. Our results show that SDA has an effect that can be observed despite leaving other influential factors uncontrolled, demonstrating the importance of the costs of digestion to the everyday energy balance of these organisms.

## Graphical abstract

We measured the metabolic costs of digestion of crabs in the field to determine its impact relative to the many other factors that can influence metabolic rates. We show that the costs of digestion are detectable, but play a relatively minor role in the overall energetic costs of the European green crab.

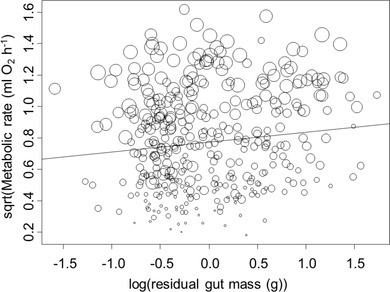

## 1 Introduction

Food consumption influences both energy intake and energy expenditure of individual animals (Jobling 2017). During digestion, metabolic rates rise in a process commonly known as specific dynamic action (SDA), or the heat of digestion, or the heat increment of feeding (Secor 2008; Jobling 2017). This process accounts for the energy needed to process and move food through the gut and digestive tract.

The magnitude and duration of the SDA response can vary with both meal composition and meal size. Meals with protein cause a greater increase in metabolic rate than that of carbohydrates or lipids because of their more complex structures (McGaw and Penney 2014; Secor 2008). Consequently, the strength of the SDA response can change due to different dietary preferences among different species and even across individuals within the same species (Secor et al. 2006; Secor 2008; Fu 2007). The impact of meal size is evident across a broad range of taxa. For example, in the catfish, *Silurus meridionalis* , the metabolic scope and duration of peak metabolic rate both increase with meal size (Fu et al. 2006). The same correlation is evident among other species including the South American rattlesnake, *Crotalus durissus* (Andrade et al. 1997), the tiger salamander, *Ambystoma tigrinum tigrinum* (Secor and Boehm 2006), across multiple species of frog (Secor et al. 2006), and across multiple species of crustaceans (McGaw and Curtis 2013).

To enhance precision, SDA studies are often conducted in the laboratory where meal size, meal composition, body size, reproductive state, individual health, temperature, and other variables can be controlled. However, the influence of these various factors can be substantial under normal field conditions. Exploring the influence of these factors in situ can contribute to understanding field metabolic rates, which has been identified as a key question in understanding the organismal responses to changing or disturbed environments (McHuron et al. 2022).

Crustaceans are an important group of aquatic consumers that often have important ecosystem impacts via consumption and modification of the environment through digging or other activities (Weis 2010). The impacts of meal size and body size on SDA have been investigated in crustaceans previously. A lab‐based study on the isopod *Ligia pallasii* found a significant relationship between increasing meal size and higher postprandial mass‐specific metabolism (Carefoot 1990). A second lab‐based study found that larger meal sizes significantly increased the duration of postprandial elevated metabolism in four out of six decapod species when comparing the smallest to largest meals (McGaw and Curtis 2013).

As with other organisms, several factors may influence SDA and metabolism in crustaceans in general. For instance, larger individuals may experience greater SDA because of their larger digestive machinery (Secor and Faulkner 2002; Luo and Xie 2008; Boyce and Clarke 1997; Gillooly et al. 2001). Other important factors that may influence metabolism in general include the amount of lipid storage in the hepatopancreas (Griffen 2017), the stage of the molt cycle (DeVries et al. 2015), limb loss and regeneration (Griffen et al. 2023; Fletcher et al. 2022), reproductive state (Smith et al. 2022), and parasite infection (Figueroa et al. 2019). The effects of each of these factors on metabolic rates could potentially obscure or overwhelm SDA effects under natural conditions.

The European green crab, *Carcinus maenas* , is a model organism for ecophysiological studies (Weihrauch and McGaw 2024). It has widely spread through invasions of littoral and sublittoral locations across the globe (Young and Elliott 2019) and has had substantial predatory impacts on invaded habitats (Frederich and Lancaster 2024; Grosholz and Ruiz 1996). The broad invasiveness and environmental impacts of *C. maenas* have also spurred an interest in its energetics, though studies on the effect of meal size on SDA in *C. maenas* are minimal. A single lab‐based study found an increase in the duration of elevated oxygen consumption during digestion of larger meals (Houlihan et al. 1990), but did not find an increase in metabolic scope (i.e., magnitude of the SDA response) with meal size.

Juvenile and large adult *C. maenas* of both sexes are found distributed broadly across intertidal habitats (Atkinson and Parsons 1973; Authors, personal observation). Intertidal individuals commonly feed, and therefore begin digestion, during high tide (Robinson et al. 2011; McDonald et al. 2006). However, the complete digestive cycle typically lasts about 12 h (McGaw and Curtis 2024; Hopkin and Nott 1980), and intertidal individuals are thus often digesting during low tides under aerial exposure, which introduces additional variability in metabolic response dependent upon humidity, temperature, and their ability to take up oxygen under aerial conditions (Simonik and Henry 2014).

This unique intertidal context may influence the SDA response in ways not captured by lab‐based studies, especially since metabolic rates of intertidal crabs can vary throughout low‐tide aerial exposure independent of digestive processes (Griffen et al. 2024). Our research aims to address this gap by examining how the mass of food in the gut (i.e., a proxy for meal size) affects metabolic rate in postprandial *C. maenas* under field conditions when other factors (low‐tide exposure, body size, reproductive state, meal type, body condition via energy storage and/or nonlethal injury, and parasitism) are all at play but are not controlled experimentally. We test the hypothesis that after accounting for these other factors statistically to the extent possible, the postprandial metabolic rate of *C. maenas* will generally increase with the increase in mass of food in the gut, reflecting SDA that depends on meal size.

## 2 Methods

### 2.1 Collections and Metabolic Measurements

We prepared 150‐mL plastic syringes for use as metabolic chambers by sealing the tips shut with silicone and drilling a hole in the barrel for extracting gas samples. During the Summer of 2022, we collected 383 *C. maenas* by hand by overturning boulders during low tide on the shores of New Hampshire and Maine from mid‐ to upper‐intertidal regions. Immediately upon collection, we placed individual crabs into syringes and adjusted the chamber volume of the syringe depending on the size of the crab (accounted for in the calculations below), so that larger crabs were given larger volumes. We also placed 0.5 mL of water into each syringe to ensure water vapor saturation throughout the trial. We then left them in a shaded location under rocks and algae at the same tidal height where they were collected, allowing them to rest for 5 min before testing. Metabolic rates can remain elevated following a disturbance for more than 16 h (McGaw and Whiteley 2024). We therefore acknowledge that 5 min is not enough time for the metabolic rates to normalize following the stress of capture and handling. However, since elevated metabolic rates following disturbance are longer than the digestive period (Wilson et al. 2021), there is no way to both measure the postprandial metabolism of naturally foraging crabs and eliminate impacts of disturbance from capture at the same time. Despite this, since we treated all individuals the same way, the impact of disturbance was surely present (likely as elevated metabolic rates), but is present across all study animals, allowing direct comparisons within our study of the relative influence of other factors that differed across study animals, including the amount of food in the gut. However, we caution that comparisons of our results to other studies may be confounded by different impacts of disturbance across studies.

At the start of a trial, we sealed the hole in the barrel of the syringe using a septum seal (Bridge Analyzers Incorporated, model #001620). At the same time, we measured the relative humidity and the barometric pressure. Without disturbing the crabs, we discretely checked them visually every 10 min throughout the trial for activity and took temperature readings immediately adjacent to them with a BTmeter (Model 100‐AAP). We adjusted the duration of a trial based on the size of each crab (accounted for in the calculations below) so that larger crabs had shorter duration trials. The variation in both the volume of the chamber and the duration of the trial was based on preliminary trials and was used to ensure that oxygen consumption was detectable, but did not create hypoxic or hypercapnic conditions. We adjusted the trial durations and volumes based on crab size as follows: for crabs with about 20–60 mm carapace width (CW), we used syringe volumes of 70–100 mL and between 40 and 60 min experiment lengths. For crabs less than 20 mm CW, we used syringe volumes of 50–70 mL and 60–80 min experiment lengths. Precise volume and time values were incorporated into metabolic rate calculations for each individual crab.

At the conclusion of each trial, we used a multi‐gas meter (Forensics Detectors, model #FD‐600, 0.01% resolution) to measure O_2_ and CO_2_ levels within the syringe. Using a built‐in pump, gas was extracted at 0.5 L min^−1^ using a needle inserted through the sealed septum. Following gas extraction, we placed crabs into individual sample bags and submerged them in ice inside a cooler for immediate transport to a freezer where they were frozen at −20°C. Crabs were then transported on dry ice to Brigham Young University, Provo, Utah, where they were stored at −80°C until dissections.

### 2.2 Dissections

After thawing, we measured body volume by water displacement using the smallest diameter graduated cylinder that could fit the crab. The number of limbs missing, number of limbs regenerating, whether a crab was gravid, and carapace color (a proxy for relative time since molt in *C. maenas* ; Reid et al. 1997) were observed and recorded. Each crab was then dissected by removing the dorsal carapace. The cardiac and pyloric stomach were removed and placed into a previously weighed aluminum boat. The hepatopancreas was also removed and placed into a separate previously weighed aluminum boat. While removing the hepatopancreas and stomachs, any acanthocephalan parasites found were counted. We then placed the remaining body and carapace into a separate previously weighed aluminum boat. Bodies, stomachs, and hepatopancreas were dried to constant weight at a temperature of 60°C after which we measured the dry mass of each to 0.0001 g using a Mettler Toledo DualRange scale (Model number XS205).

### 2.3 Metabolic Rate Calculations

We calculated oxygen consumption using equation 4.4 from Lighton (2018):

$$\mathrm{Vol}O_{2}=\left[V\left(F_{i}O_{2}-F_{e}O_{2}\right)-F_{e}O_{2}\left(\mathrm{Vol}H_{2}O\right)\right]/\left[1-F_{e}O_{2}\left(1-\mathrm{RQ}\right)\right]$$

where VolO_2_ is the volume of oxygen consumed; *V* is the volume of gas in the syringe; F_i_O_2_ is the initial fractional concentration of oxygen in the syringe (i.e., atmospheric concentration); F_e_O_2_ is the ending fractional concentration of oxygen in the syringe; VolH_2_O is the change in volume of water vapor in the syringe; RQ is the respiratory quotient, which is generally given a value between 0.7 and 1.0, representing the ratio between CO_2_ production and O_2_ consumption (Lighton 2018).

We calculated the volume of gas in the syringe (*V*) by taking the total assigned volume of the syringe and subtracting the volume of the crab, which was measured during dissections. We determined the VolH_2_O using the relative humidity, barometric pressure, and mean temperature measured during each trial and using calculators found at www.respirometry.org. For the RQ value, we assigned a value of 0.85 given that *C. maenas* is omnivorous (McGaw and Curtis 2024). This middle‐of‐the‐road value limits potential error from unknown actual diet type being digested to 3% (Vleck 1987). We then converted oxygen consumption to a rate by dividing VolO_2_ by the duration of the trial to yield VolO_2_ h^−1^. Finally, we adjusted metabolic rates to standard temperature and pressure using equation 2.1 from Lighton (2018).

### 2.4 Statistical Analyses

Studies commonly assess SDA responses using mass‐specific SDA and SDA coefficients, which both commonly suffer from the incorrect assumption that SDA scales linearly with body mass (Beaupre 2005). We avoided these assumptions in our analysis by directly using the mass of food in the gut and incorporating body mass as a covariate in tests for a nonlinear SDA response.

Before analysis, a single outlier was removed, likely from a dying crab, which showed extremely low metabolic rates for its body size. Graphical analysis of our data (qq plots, residuals vs. fitted plots, scatterplots, and histograms) revealed that the metabolic rate data were not normally distributed and body mass data were heteroskedastic. Histogram comparisons of log‐, square root‐, and ¼‐transformed data showed that square root–transformed metabolic data and log‐transformed residual gut mass data fit model assumptions best. These transformations were therefore used for analysis.

We used linear regression models with metabolic rate as the response variable. We initially fit a full model with the following predictor variables based on an ecological understanding of the impact each has on metabolism: crab sex, crab dry mass, residual gut dry mass (after accounting for body mass since larger crabs should be expected to have larger gut masses), a squared term for crab dry mass (to account for nonlinearity), a squared term for residual gut dry mass (to account for nonlinearity), whether a crab was gravid, hepatopancreas dry mass, mean temperature during metabolic trials, carapace color (using the scale provided by Young et al. 2017 as a proxy for time since molt, as *C. maenas* shifts from green to red with time since molt), number of limbs missing, number of limbs regenerating, and number of acanthocephalan parasites. We did not a priori include interaction terms between predictors because we had no ecological reason to expect interactions. In addition to this full model, we also produced a set of reduced models by sequentially removing predictors to produce all possible reduced models (i.e., all possible combinations of the predictors in the full model described above). We then compared these possible candidate models using Akaike information criterion (AIC) to determine which model provided the best fit to the data (Akaike 1974). We determined the best‐fitting model as the model with the lowest AIC and a delta AIC of at least 2 for the next best‐fitting model.

## 3 Results

**FIGURE 1:**
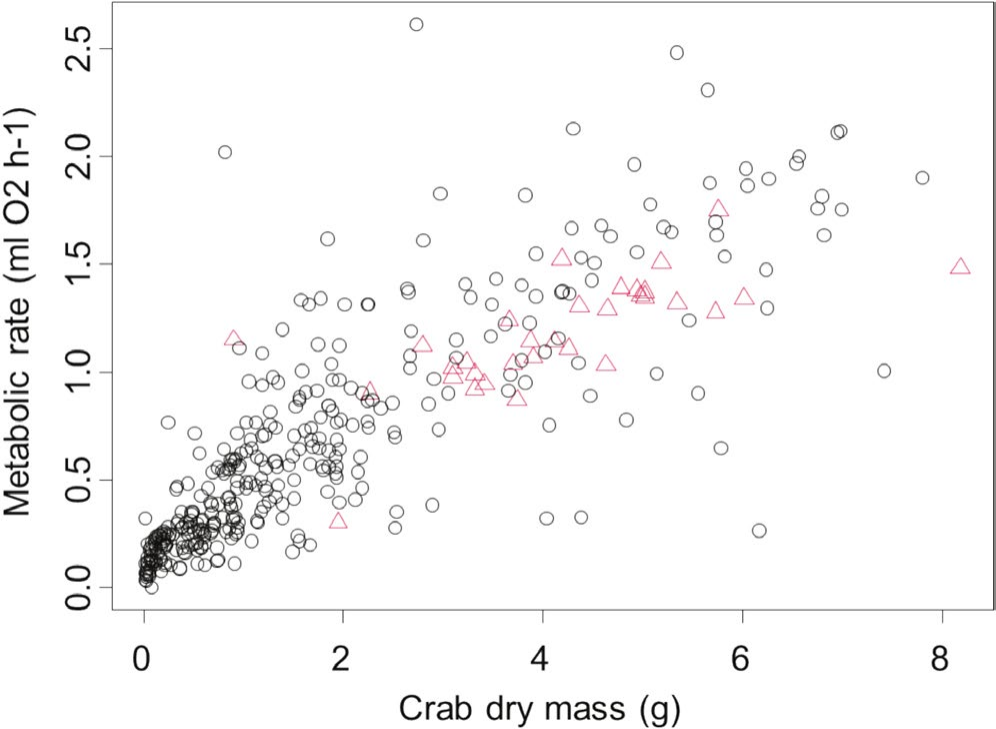
Metabolic rate as a function of crab dry mass in the European green crab *Carcinus maenas* , demonstrating the impacts of dry body mass (*x*‐axis). Red triangles represent gravid crabs.

We found that metabolic rate was influenced by several of the factors measured here. (All model parameters and confidence intervals presented below are back transformed since metabolic rates were square root transformed to meet the model assumptions of normality.) Specifically, we found that metabolic rate initially increased by 0.287 mL O_2_ h^−1^ (95% CI: 0.065–0.102) for each 1 g increase in body mass (*t* = 17.58, *p* < 0.001, Figure 1); however, this increase was nonlinear, saturating slightly at higher body masses (squared term *t* = −9.85, *p* < 0.001, Figure 1). We also found that metabolic rate was 0.0695 mL O_2_ h^−1^ lower (95% CI: 0.0003–0.0177) for crabs that were gravid than for crabs that were not gravid (*t* = −2.15, *p* = 0.032, Figure 1), a pattern that is only discernible when body size is accounted for, since most gravid crabs were relatively large and therefore had relatively high absolute metabolic rates compared to nongravid crabs. We also found that metabolic rate decreased with the mass of the hepatopancreas, but this relationship was not statistically significant (*t* = −1.66, *p* = 0.098).

**FIGURE 2:**
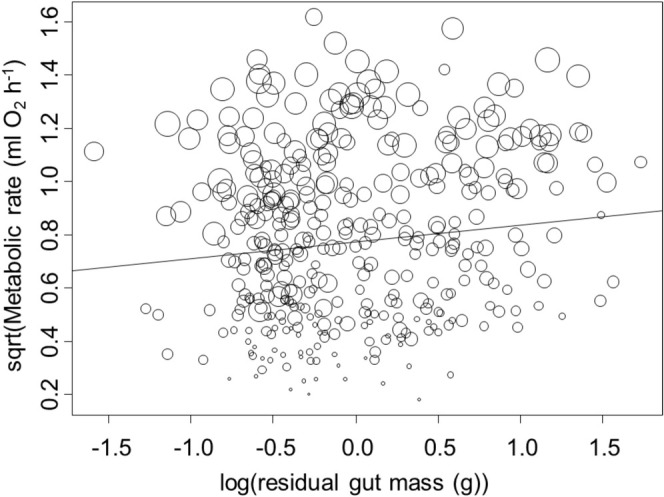
Metabolic rate as a function of residual gut mass (*x*‐axis) in the European green crab *Carcinus maenas* . Relative body mass is shown by increasing circle size. Regression line shows increasing metabolic rate with residual gut mass.

Even with the complicating impacts of body mass and reproductive state described above, the impacts of the mass of food in the gut on SDA were evident, although small. We found that metabolic rate increased by 0.062 mL O_2_ h^−1^ (95% CI: 0.001–0.008) for each 1 g increase in residual gut mass (*t* = 4.51, *p* < 0.001, Figure 2). No other parameters were included in the best‐fitting model, indicating that sex, temperature, time since molt, number of limbs missing or regenerating, and parasite infection had no detectable impact on metabolic rate after body size, reproductive state, energy storage in the hepatopancreas, and mass of food in the gut were accounted for. The best‐fitting model that included these variables explained 74% of the variation in metabolic rates, and the delta AIC of the next best‐fitting model was 5.387.

## 4 Discussion

This study explored the relationship between mass of food in the gut and metabolic rate in the European green crab *C. maenas* under conditions experienced in the field where several other factors simultaneously had the potential to influence metabolism. Our findings support the hypothesis that even when other confounding factors were present, including differences in body mass, energy storage, reproductive state, time since molt, injury status, infection by parasites, and temperature, the metabolic increases with increasing mass of food in the gut were detectable. Although field conditions lack control over digestion times, meal contents, temperature, point in the tidal cycle (Griffen et al. 2024), and other variables that lab‐based experiments can monitor and control (Dal Pont et al. 2022; McGaw and Whiteley 2012), our results demonstrate the role of mass of food in the gut under normal conditions where meal size varies together with all of these other sources of metabolic variation.

The relationship between food mass in the gut and metabolic rate found in our results, albeit minor compared to the impact of body size, is consistent with that of previous studies. Houlihan et al. (1990) identified a significant positive correlation between meal size and oxygen consumption in *C. maenas* . Additionally, McGaw and Curtis (2013) demonstrated similar patterns across other decapod species. An important difference between these studies and our study is that these previous studies based meal size on preprandial food mass and we based meal size on postprandial (likely partially digested) food mass. Differences in the mass of food in the gut measured here may therefore reflect either differences in meal size or differences in time since the meal was consumed (i.e., two crabs with different masses of food in the gut may have consumed the same size meal but at different times in the past). The full SDA response includes both the magnitude of the metabolic increase (measured here) and the duration of that increase (not measured here). Hence, we do not show the impact of meal size on SDA but rather show the impact of as yet undigested food residing in the gut on the magnitude of the SDA response. The full SDA response would require measuring metabolism during the complete digestion of the consumed food. However, it was not possible to do this while also measuring the mass of food in the gut. Nevertheless, our results demonstrate a detectable increase in metabolism with the mass of food that is currently being digested, even in the face of several other factors known to influence metabolism.

There were several additional factors that we measured, but that were not included in the best‐fitting statistical model. While not included in the best‐fitting model, many of these are known to influence metabolic rates in this or other crab species, including limb loss/regeneration (Griffen et al. 2023; Fletcher et al. 2022), parasite infection (Figueroa et al. 2019), molt stage (DeVries et al. 2015), and energy storage in the hepatopancreas (Griffen 2017). The reason that these other factors did not have a significant influence on metabolism here is unknown, but could be attributed to additional variability associated with performing these measurements in the field, to species‐specific differences between *C. maenas* and other previously studied species, to interactions between multiple factors that canceled each other out, or it may simply have been that minor impacts were drowned out by factors with stronger impacts, such as body size.

Our approach demonstrates how to measure a single factor while allowing others to vary naturally. While our study specifically focused on SDA while allowing other factors to vary, a similar approach could be used to focus on any factor under natural conditions to examine its impacts relative to other naturally occurring determinants of metabolic rate. Understanding the field metabolic rates of organisms and the relative importance of factors that influence those metabolic rates is essential for understanding how energy limitations stimulate tradeoff and constrain life history characteristics. Although this study did not directly estimate the field metabolic rate (i.e., crab movements were still physically constrained by the experimental chambers used and crabs likely experienced modified metabolic rates due to the stress of capture and handling), understanding the combination of factors in the field that can influence metabolism brings us a step closer to understanding the field metabolic rate of crabs.

In *C. maenas* , the complete digestive cycle typically lasts about 12 h (McGaw and Curtis 2024; Hopkin and Nott 1980), and feeding often occurs twice daily during high tides (Robinson et al. 2011). Effects of elevated metabolism due to SDA may likely begin upon consumption, but as our study shows, these impacts will continue into low‐tide periods as digestive processes continue. Thus, 12‐h digestion times and twice‐daily feeding frequencies suggest that individuals should often (i.e., continuously during active feeding periods of the year) be influenced by the energetic demands of SDA. Although the impact of digesting food in the gut is minor compared to other things like body size, its impact is still detectable and should be relatively constant.

## Author Contributions

**David L. Neu:** formal analysis (equal), writing – original draft (lead). **Laura S. Fletcher:** data curation (equal), writing – review and editing (supporting). **Mikayla Bolander:** data curation (equal), writing – review and editing (supporting). **Vibalia Raj:** data curation (equal), writing – review and editing (supporting). **Bailey N. Marlow:** data curation (equal), writing – review and editing (supporting). **Blaine D. Griffen:** conceptualization (equal), data curation (equal), formal analysis (equal), funding acquisition (equal), investigation (equal), methodology (equal), project administration (equal), supervision (equal), visualization (equal), writing – review and editing (lead).

## Conflicts of Interest

The authors declare no conflicts of interest.

## Supporting information


Appendix S1

## Data Availability

All data used in this paper are included as Supporting Information.
